# Cancer risk among transgender adults: A growing population with unmet needs

**DOI:** 10.1111/aogs.14686

**Published:** 2023-10-16

**Authors:** Sarah S. Jackson, Anne Hammer

**Affiliations:** ^1^ Division of Cancer Epidemiology and Genetics National Cancer Institute Bethesda Maryland USA; ^2^ Department of Clinical Medicine Aarhus University Aarhus Denmark; ^3^ Department of Obstetrics and Gynecology Gødstrup Hospital Herning Denmark

Globally, an estimated 1% of the population identifies as transgender, with 2%–3% of individuals under the age of 25 identifying as transgender or gender diverse.^1^ Transgender is an umbrella term for individuals whose gender identity differs from their sex assigned at birth. Nonbinary individuals are those whose identity falls outside of the gender binary (eg man or woman) and include genderqueer, gender nonconforming, agender, and other identities.^1^ Some transgender individuals may take gender affirming hormones or undergo gender affirmation surgery (collectively known as gender affirming care) so that their outward gender expression matches their internal gender identity. It is important to note that gender affirming care is very individualized, with some people choosing to postpone or forgo some treatments to preserve fertility, or for personal or financial reasons. Some individuals may choose not to undergo a physical transition^1^ or they may feel that neither the masculine or feminine gender expression matches their identity.^1^

Cancer is an understudied topic in transgender health due to paucity of available data.^2^ Gender identity is not collected in the census, cancer registries, or most population‐based cohort studies.^2^ Therefore, research on cancer in this population has been limited to a few case reports.^1^ The incidence of cancers among transgender persons may be different than that of cisgender persons for reasons related to sex hormones and gender affirming surgery, differential exposure to viral infections, and increased exposure to stigma and discrimination. A summary of available evidence is presented in the Box 1.

BOX 1 **Summary of available evidence and implications for clinical practice**

**Table:** 

	Transgender women/nonbinary people assigned male at birth	Transgender men/nonbinary people assigned female at birth	Implications for clinical practice
Breast cancer	Risk increased	Risk decreased	Mammography may be warranted for both groups Possible increased gender dysphoria from screening/treatment Be aware of barriers to screening/treatment
Cervical cancer	N/A	Risk may be increased; more research is needed	Follow current guidelines Possible increased gender dysphoria from pelvic examination Barriers to screening/treatment
Prostate cancer	Risk decreased	N/A	Follow current guidelines Be aware that estrogen may increase tumor aggressiveness Possible increased gender dysphoria from rectal examinations and treatment Barriers to screening/treatment
Testicular cancer	No change in risk	N/A	Possible gender dysphoria related to examinations Barriers to screening/treatment
Non‐reproductive cancers	More research is needed	More research is needed	Follow current guidelines as applicable Barriers to screening/treatment

Increased circulating estradiol levels from both endogenous and exogenous sources are associated with breast cancer risk in cis women; however, the evidence on the risk of breast cancer in transgender women is mixed. An analysis among United States (US) veterans found no increased incidence of breast cancer among 5135 transgender women who used estrogen compared to cisgender men.^3^ On the other hand, a Dutch study found an elevated risk of breast cancer among 2260 transgender women on estrogen therapy compared to a Dutch cisgender male population (Standardized Incidence Ratio (SIR): 46.7). The risk of breast cancer among transgender women was lower than that of a Dutch cisgender female population (SIR: 0.30).^4^ Among transgender men who have undergone bilateral mastectomy, the risk of breast cancer is decreased compared to cisgender women, yet still significantly elevated when compared to cisgender men.^4^ Importantly, the age of breast cancer diagnosis was lower in both transgender women (52 years) and transgender men (46 years) than the average age of diagnosis for Dutch cisgender women (61 years).^4^ Although more studies are needed on breast cancer risk, these findings highlight the importance of continued breast cancer screening for transgender men who have not had bilateral mastectomy.

As androgens are thought to be the primary driver of prostate cancer it has been hypothesized that transgender women would have a lower risk of prostate cancer as many individuals use hormone therapy along with orchiectomy to reduce testosterone levels. Indeed, a study conducted in the Netherlands of 2260 transgender women who had undergone gender affirming care found a lower prostate cancer risk in transgender women compared to cisgender men (SIR: 0.20).^5^ Indeed, androgen‐suppressing drugs are used for the treatment for prostate cancer. However, there is concern that the use of estrogen therapy in transgender women may lead to more aggressive tumors and poorer prognosis as estrogens can be pro‐tumorigenic. The same Dutch cohort did not find the risk of testicular cancer to be altered in transgender women compared to cisgender men.^6^

Transgender persons may be at higher risk of certain cancers compared to cisgender persons, in part, due to an increased prevalence of infections like HIV.^7^ The global prevalence of HIV among transgender women is 19%^7^ largely due to social stigma and economic marginalization leading to commercial sex work in some countries.^1^, ^7^ The prevalence of human papillomavirus (HPV) in transgender populations is unknown, though some evidence suggests that it is similar in transgender men and cisgender women.^8^ Recent results from a treatment trial for anal cancer among people with HIV indicate that transgender people may have a high burden of HPV‐related anal cancer.^9^

Transgender persons may be at higher risk for cancers due to coping mechanisms related to discrimination, stigma, and social isolation.^1^, ^10^, ^11^ In a US study of transgender persons, 63% of the respondents reported they had experienced a serious act of discrimination due to their trans status.^10^ These discrimination events including loss of job, housing or education, physical or sexual assault, alienation from family, incarceration, and denial of medical services.^10^ These events can have a major impact on quality of life and financial and emotional stability. Stigma, discrimination, and victimization have been identified as strong mediators in the relationship between gender identity and all the health outcomes among older transgender adults.^11^ The accumulation of these daily stressors can result in engagement in harmful coping mechanisms leading to poor health outcomes.^11^ A growing body of literature suggests that transgender people have a higher prevalence of alcohol and tobacco use, and lack of physical activity, all significant risk factors for cancer.^12^, ^13^ Large scale, longitudinal studies among transgender people are needed to elucidate the impact these stressors have on cancer either directly or indirectly through unhealthy behaviors.

Discrimination in healthcare settings has been documented as a major barrier to transgender people accessing care in both the US and the UK.^14^, ^15^ These surveys of transgender persons found that a majority of individuals experienced transphobia in medical settings.^14^, ^15^ A significant proportion of patients have reported being refused health care of any kind by a clinician for being transgender with some even experiencing violence by clinic staff.^14^, ^15^ More than 50% of transgender persons in England have reported avoiding healthcare visits when ill due to these experiences.^14^ These barriers also decrease the likelihood transgender persons will obtain preventative services, such as smoking cessation or cancer screening, leading to increased cancer disparities in this population.^1^ Thus, it is important that clinicians be aware of the cancer risks in transgender populations, but systems level changes may also be needed to ensure that transgender people are invited for routine screening, and that cervical samples from individuals with a male personal identification number are processed similarly to samples collected from cis women. Further, transgender people may forgo cancer screening as it can exacerbate feelings of gender dysphoria (discomfort with anatomy that conflicts with gender identity) for many.^8^ Potential solutions may include the establishment of transgender‐inclusive screening centers or self‐testing for cervical cancer.^8^

Medical discrimination, among other factors, may even increase cancer outcome disparities. A study from the US reported that transgender adults with cancer had worse outcomes than cisgender adults with cancer.^16^ Transgender persons were more likely to be diagnosed with some cancers at later stages, less likely to receive treatment for cancer, and had an increased risk of death for prostate cancer, non‐Hodgkin lymphoma, and bladder cancer.^16^

In summary, the prevalence of individuals reporting and seeking treatment for gender incongruence is growing globally. Therefore, there is an urgent need to explore cancer risk and cancer mortality in transgender persons as the incidence of certain cancers may be altered from that of cisgender persons. Given that many cancer risk factors are increased in this population, greater efforts are needed to ensure transgender adults undergo routine screening at the same rates as cisgender people. Finally, documenting transphobia and discrimination in healthcare is needed to provide interventions to clinicians in order to improve access to cancer prevention and care for all transgender individuals.

## CONFLICT OF INTEREST STATEMENT

The authors confirm that there are no conflicts of interest.
